# Potential therapeutic manipulations of the CXCR3 chemokine axis for the treatment of inflammatory fibrosing diseases

**DOI:** 10.12688/f1000research.26728.1

**Published:** 2020-10-05

**Authors:** Morgan K. Groover, Jillian M. Richmond

**Affiliations:** 1 Department of Dermatology, University of Massachussetts Medical School, Worcester, MA, 01605, USA

**Keywords:** CXCR3, CXCL9, CXCL10, CXCL11, fibrosis, fibroblast, pericyte, endothelial cell

## Abstract

Chemokines play important roles in homeostasis and inflammatory processes. While their roles in leukocyte recruitment are well-appreciated, chemokines play additional roles in the body, including mediating or regulating angiogenesis, tumor metastasis and wound healing. In this opinion article, we focus on the role of CXCR3 and its ligands in fibrotic processes. We emphasize differences of the effects of each ligand, CXCL9, CXCL10 and CXCL11, on fibroblasts in different tissues of the body. We include discussions of differences in signaling pathways that may account for protective or pro-fibrotic effects of each ligand in different experimental models and *ex vivo* analysis of human tissues. Our goal is to highlight potential reasons why there are disparate findings in different models, and to suggest ways in which this chemokine axis could be manipulated for the treatment of fibrosis.

## Introduction: understanding CXCR3’s typical and atypical functions

Chemokine receptors are a subgroup of class A G-protein coupled receptors (GPCRs) that are relatively conserved across eukaryotes ^1^. They bind to chemokine ligands, a special class of 8–10kDa chemotactic cytokines, which are classified based on their amino acid structure (e.g. CC, CXC, or CX3C) ^2^. With a few exceptions, most ligand-receptor relationships are promiscuous, meaning that a single chemokine receptor has multiple ligands and a single chemokine can bind to multiple receptors. As of now, there are 18 known chemokine receptors with Gαi-dependent chemotactic activity, and 5 atypical (non-chemotactic, recycling or scavenging) chemokine receptors in humans. Many chemokines are considered inflammatory, as they recruit leukocytes during inflammatory responses. However, there are also homeostatic chemokines that are important for immune cell maturation, tissue development, and angiogenesis. Homeostatic chemokines often exhibit tissue tropism, providing signals for recirculating immune cells, paracrine signals for cells that comprise tissues, and even tumor growth and metastasis ^3^.

CXCR3 is typically considered to be an inflammatory chemokine receptor because it is expressed by leukocytes that migrate towards interferon-induced ligands to sites of tissue inflammation ^4^. However, CXCR3 is also expressed on non-hematopoietic cells including endothelial cells, where it plays roles in promoting or inhibiting angiogenesis, and fibroblasts, in which it mediates wound healing responses.

There are several examples of diseases where inflammation precedes or is admixed with fibrosis, including infectious diseases (e.g. schistosomiasis, tuberculosis), cancers (e.g. pancreatic cancer, post-irradiation breast cancer) and autoimmune diseases (e.g. hepatitis, pulmonary fibrosis in scleroderma and skin fibrosis in morphea). Hallmarks of inflammatory fibrosis include infiltration of leukocytes; activation of endothelium; fibroblast activation, migration, proliferation and differentiation; production of collagen and other extracellular matrix proteins; and increased collagen bundle thickness and disorganization ^5^. Data from our lab and others have demonstrated that the CXCR3 chemokine axis can mediate protective or pro-fibrotic signals depending upon the context of the involved organs. In this opinion article, we will discuss potential reasons for disparate findings, and provide our opinions about how this system can be targeted therapeutically for the treatment of fibrosis.

## CXCR3 signaling pathways in leukocytes

CXCR3 has four extracellular domains that bind its ligands (CXCL9, CXCL10, and CXCL11), and four intracellular domains that mediate the receptor’s different functions. The differential involvement of CXCR3 receptor domains in ligand binding and subsequent differences in downstream signaling contribute to the complex nature of this chemokine system, which has been mapped out in leukocytes using mutational constructs and competition binding assays. Like many other GPCRs, CXCR3-mediated chemotaxis is pertussis-toxin sensitive. However, CXCR3 activates several other pathways in addition to Gα subunit proteins, which we will review below.

**Figure 1.**
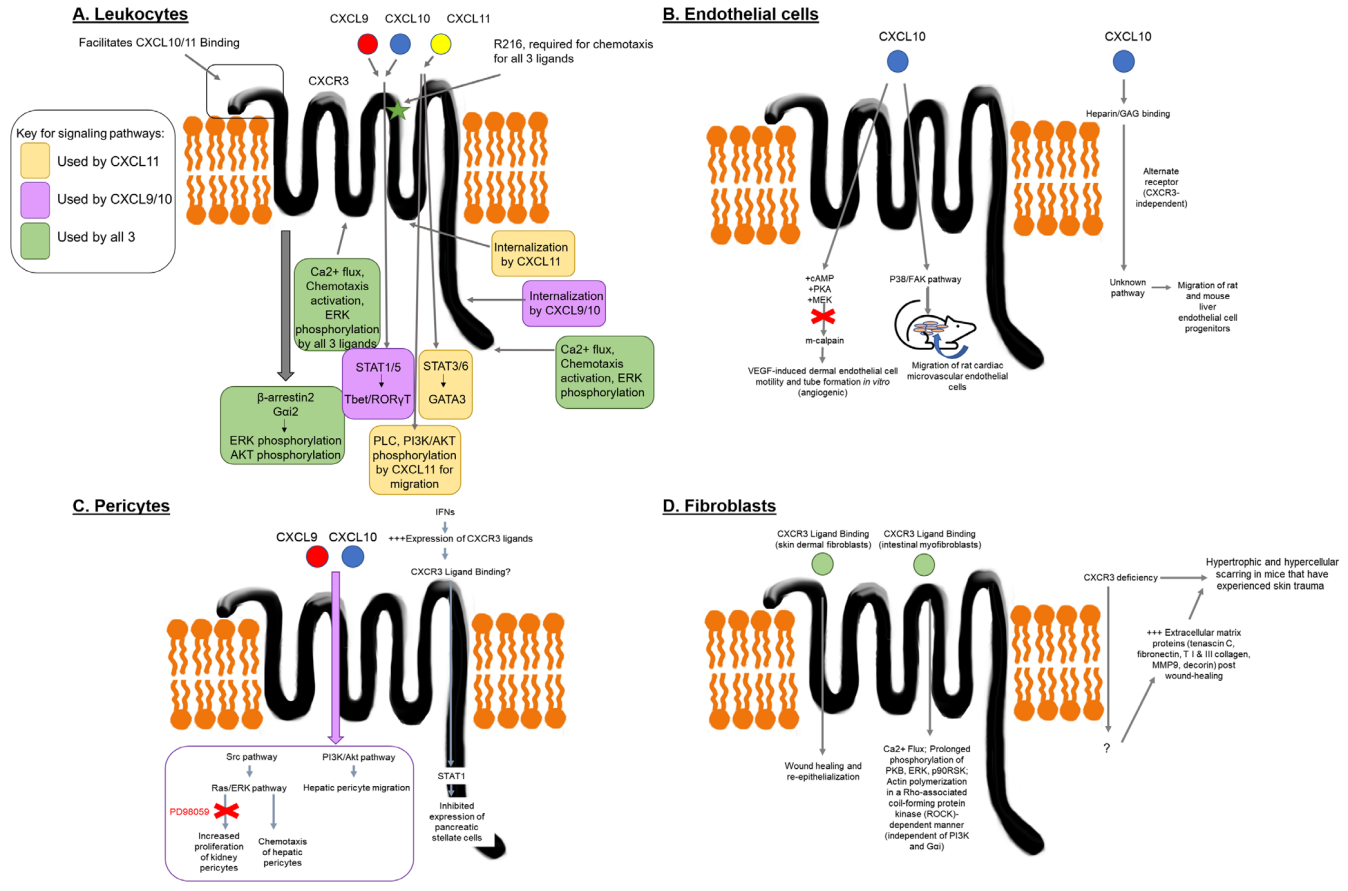
CXCR3 signaling pathways in different cell types. ( **A**) *Major CXCR3 signaling pathways in leukocytes.* CXCL9, CXCL10 or CXC11 bind to CXCR3 to mediate chemotaxis and T cell skewing. Different domains facilitate ligand binding, with the N terminus of CXCR3 facilitating binding of CXCL10 and CXC11. R216 in the second extracellular loop (green star) is required for chemotactic responses for all 3 ligands. All three ligands can induce calcium flux, pERK, and pAKT, though CXCL9/10 require Gαi2 for pERK and β-arrestin2 for pAkt. CXCL11 can activate PLC and PI3K/AKT to mediate migration independent of Gαi. Internalization of CXCR3 induced by CXCL9/10 requires the C terminus, whereas CXC11 requires the 3rd intracellular loop. CXCL9/10 can activate STAT1/5 to enforce Tbet/RORγT expression, whereas CXCL11 activates STAT3/6 to enforce GATA3 expression. ( **B**) *CXCR3-dependent and independent signaling pathways in endothelial cells.* CXCL10 activates cAMP, PKA and MEK in dermal endothelial cells to inhibit m-calpain and dampen angiogenesis. In cardiac microvascular endothelial cells, CXCL10 activates the p38/FAK pathway to induce migration, but not proliferation. CXCL10 also exerts effects on endothelial cells independently of CXCR3, but in a manner that requires GAG binding. ( **C**) *CXCR3 signaling in pericytes.* Pericytes activate Src, Ras/ERK and PI3K/AKT pathways downstream of CXCR3, which mediate chemotactic responses. Kidney pericytes exhibit increased proliferation downstream of CXCL9/10, which is ERK-dependent (inhibited by PD98059). Interferons inhibit proliferation of pancreatic stellate cells via STAT1, though it is unclear whether this response is via CXCR3 ligands. ( **D**) *Fibroblast responses to CXCR3 ligands.* Intestinal myofibroblasts exhibit calcium flux and phosphorylation of PKB, ERK, p90RSK induced by all three ligands. All three ligands induce actin polymerization in a Rho-associated coiled coil-forming protein kinase (ROCK)-dependent manner that is independent of PI3K and Gαi. Signaling in skin dermal fibroblasts has not been fully mapped, though CXCR3 deficiency leads to hypertrophic and hypercellular scarring in mice via increased extracellular matrix proteins, including tenascin C, fibronectin, type I & III collagen, MMP9 and decorin. Color key: CXCL9 = red; CXCL10 = blue; CXCL11 = yellow; CXCL9/10 = purple; CXCL9/10/11 = green.

CXCR3 binding and activation requires ligand interactions with at least one sulfated tyrosine in the N terminus and an interaction with amino acid residue R216 in the second extracellular loop ^6^. The proximal 16 amino acid residues of the N terminus are required for CXCL10 and CXCL11 binding and activation, but not CXCL9 activation. R216 in the second extracellular domain plays no role in CXCL10 or CXCL11 binding or ligand-mediated internalization, but this residue is necessary to activate chemotaxis by all three CXCR3 ligands ^7^. Both the DRY site, which encompasses the R216, and the CXCR3 carboxyl terminus are essential for CXCL9-, CXCL10-, and CXCL11-induced chemotaxis, calcium mobilization, and Erk phosphorylation ( Figure 1A).

CXCR3 ligands selectively activate different receptor internalization pathways via β-arrestin and Gαi family members. Differences in signaling pathway activation by each ligand is called biased agonism ^8, 9^. CXCL10-induced receptor internalization relies on the CXCR3 carboxyl terminus, dynamin and β-arrestin1 ^10^. CXCL11 is the most potent inducer of CXCR3 internalization ^11^, and dominant negative dynamin and β arrestin 1 are unable to impede internalization ^10^. The third intracytoplasmic loop is required for maximal CXCL11-induced internalization ( Figure 1A).

In T cells, Gαi2 is required for mediating CXCR3 ligand signaling, whereas Gαi3 limits activation of this signaling pathway ^12^. Western blotting of peripheral blood leukocytes stimulated with CXCR3 ligands demonstrated that CXCL11 and, to a lesser extent, CXCL9/10 induce dose- and time-dependent phosphorylation of p44/42 MAPK (ERK) and Akt that is prevented by pertussis toxin treatment (inhibition of Gα subunit binding) ^13^. However, inhibition of MEK/ERK (U0126 or PD98059) does not prevent CXCL11-mediated chemotaxis, whereas PLC inhibition (U73122), PI3K (wortmannin) or, to a lesser extent, AKT inhibition (LY294002) does abrogate or reduce human T cell migration, respectively. Akt activation in human T cells was recently reported to be dependent upon β-arrestin2 ^14^.

There is increasing evidence that chemokine receptors can mediate JAK/STAT signaling, which has typically been attributed to common gamma chain cytokine signaling ^15^. While JAK activation downstream of CXCR3 has not yet been studied, STAT activation in response to incubation with CXCL9/10/11 has been assessed in T cell cultures: addition of recombinant CXCL9/10 activates STAT1/STAT5 to promote Th1 and/or Th17 differentiation via Tbet/RORγT expression, whereas CXCL11 activates STAT3/STAT6 via GATA3 expression to augment regulatory function ^16^.

## CXCR3 signaling pathways in vascular endothelial cells, smooth muscle cells, pericytes and fibroblasts

CXCR3 is also expressed by some non-hematopoietic cells, including endothelial cells, smooth muscle cells and fibroblast subsets. In endothelial cells, CXCR3 ligands mediate pro- or anti-angiogenic signals depending upon the model and tissue of origin. CXCL10 inhibits VEGF-induced dermal endothelial cell motility and tube formation *in vitro* via cAMP, PKA and MEK inhibition of m-calpain; this likely serves as a way to inhibit angiogenesis late in the wound healing process ^17^. CXCL10 exhibits angiostatic properties in non-small cell lung cancer and idiopathic pulmonary fibrosis ^18, 19^. CXCL10 is also able to induce angiostatic effects by binding glycosaminoglycans (GAGs) independently of CXCR3 ^20^. However, CXCL10 induces migration, but not proliferation, of rat cardiac microvascular endothelial cells via the p38/FAK pathway ^21^ and rat and mouse liver endothelial cell progenitors through an unknown pathway ^22^ ( Figure 1B).

Pericytes are contractile cells on capillaries and post-capillary venules in tissues that play integral roles in tissue healing and remodeling ^23^. Examples include hepatic stellate cells in the liver, glomerular mesangial cells in the kidney and pancreatic stellate cells. Pericytes express CXCR3, and activate the Src, Ras/ERK, and PI3K/Akt pathways downstream of CXCR3 ligand binding ^24^. Inhibitor studies indicate that Src and subsequent Ras/ERK activation are required for chemotaxis of hepatic pericytes, which is in direct contrast to observations in T cells. PI3K/Akt also plays an important role in hepatic pericyte migration as evidenced by abrogation of CXCL10 migration in the presence of wortmannin or LY294002. In addition to chemotaxis, kidney pericytes exhibit increased proliferation downstream of CXCL9/10, which is ERK-dependent (inhibited by PD98059). Unlike hepatic pericytes, kidney pericytes exhibit a second wave of ERK phosphorylation following incubation with CXCL10. CXCR3 signaling in pancreatic stellate cells has not been as extensively mapped, but they respond to PDGF by activating Src-JAK2-STAT3 ^25^. Interferons (IFNs), which drive expression of CXCR3 ligands, inhibit proliferation of pancreatic stellate cells via STAT1 ^26^ ( Figure 1C).

CXCR3 plays a homeostatic role in wound healing and re-epithelialization responses by fibroblasts ^27– 29^. CXCR3 deficiency leads to hypertrophic and hypercellular scarring in mice that have experienced skin trauma ^30, 31^. While CXCR3-mediated signaling in skin fibroblasts has not been well-characterized, the consequence of loss of signaling during wound healing includes increases in extracellular matrix proteins including tenascin C, fibronectin, type I & III collagen, MMP9 and decorin 180 days post-wounding compared to WT controls ^31^.

Studies of CXCR3 signaling downstream of CXCL9 and CXCL10 in intestinal myofibroblasts have shown modest differences in signaling, including CXCL9/10-induced calcium flux at 10min versus 8min for CXCL11, and prolonged phosphorylation of PKB, ERK, p90RSK induced by all 3 ligands as compared to shorter phosphorylation time in peripheral blood leukocytes (e.g. 2-20min versus 1-2min) ^32^. All three ligands induce actin polymerization in a Rho-associated coiled coil-forming protein kinase (ROCK)-dependent manner that is independent of PI3K and Gαi ( Figure 1D). Further detailed signaling pathway analyses for CXCL9/10/11 signaling are warranted in non-hematopoietic cells from different organs.

## Post-translational modifications, proteolytic processing and potential alternate receptors for the CXCR3 chemokine axis

Post-translational modifications of the CXCR3 chemokine axis modulates the function and signaling ability of the ligands and their receptor. CXCL10/11 have heparin binding sites that allow it to be presented on endothelium ^33^. CXCL10 presentation by the endothelium requires oligomerization ^34^. CXCL10/11 may be citrullinated by peptidylarginine deiminase, which inhibits their ability to induce chemotaxis and calcium flux and reduces their ability to bind heparin ^35^. CXCR3 itself requires tyrosine sulfation to bind to its ligands and mediate chemotaxis ^6^.

CXCL9/10/11 are cleaved/truncated by CD26, and CXCL11 is cleaved by CD13 ^36, 37^. The CD26 truncations of CXCR3 ligands retain angiostatic activity while losing CXCR3-mediated signaling ^38^. The C’ terminus of CXCL9 can inhibit neutrophil migration via competition with CXCL8-mediated binding to heparin, heparan sulfate, and cellular GAGs, which normally facilitate adhesion to vessels and subsequent transmigration ^39, 40^. CD13 is expressed by endothelial and epithelial cells as well as fibroblasts in angiogenic tissue, but not normal tissue ^41^. Truncation of just the first two amino acids in CXCL11 by CD13 abrogates Akt and ERK phosphorylation and greatly reduces calcium flux to prevent migration of CXCR3-transfected CHO cells ^36^. Truncation of the first six amino acids still retains angiostatic activity as assessed by scratch assay of endothelial cell cultures.

There are two other isoforms of CXCR3: CXCR3-B which binds to CXCL4 and mediates angiostatic effects in cultured human endothelial cells ^42^; and CXCR3-alt which binds CXCL11 ^43^. Of note, C57BL/6 (B6) mice do not express CXCR3-B ^20^. The roles of CXCR3-B and CXCR3-alt in fibrosis have not been studied. CXCR3 can also crosstalk with CXCR4 and CXCR7 via CXCL11 and CXCL12 ^44^. CXCR4 mediates profibrotic effects in the liver, while CXCR7 mediates more homeostatic regenerative responses ^45^. CXCL9 can induce heterologous desensitization of CXCR4 to its ligand CXCL12 ^46^. Notably, autoantibodies against CXCR3 and CXCR4 correlate with increased lung and skin disease severity in scleroderma patients, though it is unclear exactly how these impact signaling ^47, 48^. CXCL11 binds to CXCR7, which is expressed on activated endothelial cells, tumor cell lines and fetal liver cells ^49^. CXCL11 ligation by CXCR7, which has an affinity of 2-5nM, does not induce calcium flux or migration; rather it promotes survival and adhesion. CXCR7 has been proposed to be a scavenger receptor for CXCL11 ^50^. CXCR7 can attenuate TGFβ signaling in the lung, though the role of CXCL11 in this process has not been studied ^51, 52^.

## Profibrotic roles of CXCR3 ligands

CXCR3 and its ligands are reported to promote fibrosis in certain disease models and organs. An important caveat to bear in mind when assessing B6 mouse models is that CXCL11 is not expressed in this strain due to a null mutation. However, CXCL9 and CXCL10 knockout mice were generated using 129 oocytes and were backcrossed to B6. Therefore, WT B6 mice express CXCL9 and CXCL10, CXCL9-/- mice express CXCL10 and CXCL11, and CXCL10-/- mice express CXCL9 and CXCL11. This means that while CXCL11 cannot be directly assessed in B6 models, insights about its function can be gleaned by comparing CXCL9-/-, CXCL10-/- and WT B6 mice.

The nephrotoxic serum nephritis model of inflammatory kidney disease, which exhibits tubulointerstitial fibrosis, is dependent on CXCR3 and CXCL9, but not CXCL10, as determined by histopathology and loss of renal function ^53^. CXCR3-/- and CXCL9-/- mice had fewer intrarenal activated T cells and macrophages, as well as fewer IgG glomerular deposits and antigen-specific IgG in serum. These data suggest that CXCR3 and CXCL9 initiate nephritis through cell-mediated events, which ultimately promote tubulointerstitial fibrosis. CXCL10-/- animals developed kidney disease similar to WT controls, indicating that any potential antifibrotic role of CXCL11 in the kidney is potentially nullified by profibrotic effects of CXCL9. Similarly, any potential profibrotic role of CXCL11 in CXCL9-/- mice may be nullified by antifibrotic effects of CXCL10. However, a Balb/c mouse model of unilateral ureteral obstruction-induced renal tubulointerstitial fibrosis was exacerbated by JAK inhibition, and STAT3 played a protective role ^54^. Several factors may contribute to the disparate findings between these models, namely whether the process is immune-mediated or obstructive nephropathy, which other signals are being disrupted by JAK inhibition, and whether all three CXCR3 ligands are present to balance pro- versus anti-fibrotic signaling ( Table 1).

**Table 1.** Summary of the effects of each CXCR3 ligand in different organ systems and models of fibrosis.

Ligand	Organ	Disease	Experimental model	Species	Effect on fibroblasts/ fibrosis?	Study outcomes	Reference(s)
**CXCL9**	**Heart**	Myocardial infarction	Spontaneous; isoproterenol- induced	Human; Rat	Proliferation & migration	Increased fibrosis following MI; cytokines released by myocardium induced expression of CXCL9 which promoted fibroblast proliferation & migration	Lin *et al.,* 2019
		Rheumatic fever	Spontaneous	Human	no direct effect shown	Increased migration of inflammatory infiltrates specifically to valves and correlated with amount of cardiac fibrosis	Faé *et al.,* 2013
		Chagas cardiomyopathy	Spontaneous; infduced	Human; Beagle dog	no direct effect shown	Increased migration of inflammatory infiltrates to the heart; polymorphism CXCL9 rs10336 CC was associated with protection from progression to severe CCC	Nogueira *et al.,* 2012
	**Kidney**	Inflammatory Kidney Disease w tubulointerstitial fibrosis	Nephrotoxic serum nephritis	Mouse	Pro-fibrotic	Pro-fibrotic: initiates nephritis through cell mediated events	Menke *et al.,* 2008
	**Liver**	Hepatic fibrosis	carbon tetrachloride- induced	Mouse	Anti-fibrotic	Angiostatic and antifibrotic via modulation of stellate cell activation and endothelial cell inhibition. May or may not influence skewing of Th1-polarized, IFN-γ-positive cells in the liver.	Sahin *et al.,* 2012, Wasmuth *et al.,* 2009
		Liver cirrhosis	Spontaneous	Human	no direct effect shown	Low levels correlated with better survival following transjugular intrahepatic portosystemic shunt	Berres *et al.,* 2015
		Hepatitis C Virus-associated fibrosis	Spontaneous	Human	dependent upon genotype	Alleles/polymorphisms of CXCL9/10/11 are associated with protection or promotion of fibrosis	Jiménez-Sousa *et al.,* 2017, Pineda-Tenor *et al.,* 2015
	**Lung**				?	not yet studied	
	**Pancreas**	Chronic pancreatitis	Trinitrobenzene sulfonic acid (TNBS) induced	Rat	Anti-fibrotic	Attenuates fibrogenesis in vivo; has antifibrotic effects in vitro	Shen *et al.,* 2013
	**Skin**	Morphea	Spontaneous	Human	Pro-fibrotic	Serum levels are correlated with disease activity	O'Brien *et al.,* 2017; Mertens *et al.,* 2018
	**Multiorgan**	Systemic scleroderma	Spontaneous	Human	no direct effect shown	Increased levels are documented in disease	Hasegawa *et al.,* 2011; Liu *et al.,* 2013; Rabquer *et al.,* 2011
**CXCL10**	**Heart**	Chagas cardiomyopathy	Spontaneous; induced	Human; Beagle dog	no direct effect shown	Increased migration of inflammatory infiltrates to the heart; polymorphism CXCL10 rs3921 GG was associated with protection from progression to severe CCC	Nogueira *et al.,* 2012
	**Kidney**	Inflammatory Kidney Disease	Nephrotoxic serum nephritis	Mouse	no direct effect shown	Not fully understood but seems to be dispensable for pathology	Menke *et al.,* 2008
	**Liver**	Hepatic fibrosis	carbon tetrachloride- induced	Mouse	Pro-fibrotic	CXCL10 prevents NK cells from inactivating hepatic stellate cells	Hintermann *et al.,* 2010
	**Lung**	Pulmonary fibrosis	Bleomycin induced	Mouse	Anti-fibrotic	Limits fibrosis by reducing fibroblast migration to lung tissue	Tager *et al.,* 2004; Jiang *et al.,* 2010
	**Pancreas**				?	not yet studied	
	**Skin**	Morphea	Spontaneous	Human	no direct effect shown	Serum levels are correlated with disease activity	Mertens *et al.,* 2018
	**Multiorgan**	Systemic scleroderma	Spontaneous	Human	no direct effect shown	Increased levels are documented in disease	Hasegawa *et al.,* 2011; Liu *et al.,* 2013; Rabquer *et al.,* 2011
**CXCL11**	**Lung**	Pulmonary Fibrosis	Bleomycin induced	Mouse	Anti-fibrotic	Systemic CXCL11 administration reduced pulmonary collagen deposition, procollagen gene expression, and histopathologic fibroplasia and extracellular matrix deposition in the lung. CXCR3 is not expressed on fibroblasts; CXCL11 had no direct effect on pulmonary fibroblasts.	Burdick *et al.,* 2005
		Systemic scleroderma	Spontaneous	Human	no direct effect shown	High bronchoalveolar lavage fluid CXCL11 correlates with less risk of developing interstitial lung disease	Cardarelli *et al.,* 2012
**CXCL4** **(binds** **CXCR3-B)**	**Liver**	Hepatic fibrosis	carbon tetrachloride- induced	Mouse	no direct effect shown	Angiostatic: Directly interrupts VEGF signaling	Sulpice *et al.,* 2004

**Table 1 Complete References:** 1. Lin C-F, Su C-J, Liu J-H, Chen S-T, Huang H-L, Pan S-L. Potential Effects of CXCL9 and CCL20 on Cardiac Fibrosis in Patients with Myocardial Infarction and Isoproterenol-Treated Rats. J Clin Med Res [Internet]. 2019 May 11;8(5). Available from: http://dx.doi.org/10.3390/jcm8050659 2. Faé KC, Palacios SA, Nogueira LG, Oshiro SE, Demarchi LMF, Bilate AMB, et al. CXCL9/Mig mediates T cells recruitment to valvular tissue lesions of chronic rheumatic heart disease patients. Inflammation [Internet]. 2013 Aug;36(4):800–11. Available from: http://dx.doi.org/10.1007/s10753-013-9606-2 3. Nogueira LG, Santos RHB, Ianni BM, Fiorelli AI, Mairena EC, Benvenuti LA, et al. Myocardial chemokine expression and intensity of myocarditis in Chagas cardiomyopathy are controlled by polymorphisms in CXCL9 and CXCL10. PLoS Negl Trop Dis [Internet]. 2012 Oct 25;6(10):e1867. Available from: http://dx.doi.org/10.1371/journal.pntd.0001867 4. Menke J, Zeller GC, Kikawada E, Means TK, Huang XR, Lan HY, et al. CXCL9, but not CXCL10, promotes CXCR3-dependent immune-mediated kidney disease. J Am Soc Nephrol [Internet]. 2008 Jun;19(6):1177–89. Available from: http://dx.doi.org/10.1681/ASN.2007111179 5. Sahin H, Borkham-Kamphorst E, Kuppe C, Zaldivar MM, Grouls C, Al-samman M, et al. Chemokine Cxcl9 attenuates liver fibrosis-associated angiogenesis in mice. Hepatology [Internet]. 2012 May 19;55(5):1610–9. Available from: http://doi.wiley.com/10.1002/hep.25545 6. Pineda-Tenor D, Berenguer J, García-Álvarez M, Guzmán-Fulgencio M, Carrero A, Aldámiz-Echevarria T, et al. Single Nucleotide Polymorphisms of CXCL9-11 Chemokines Are Associated With Liver Fibrosis in HIV/HCV-Coinfected Patients. JAIDS Journal of Acquired Immune Deficiency Syndromes [Internet]. 2015 Apr 1 [cited 2020 Sep 23];68(4):386. Available from: https://journals.lww.com/jaids/fulltext/2015/04010/Single_Nucleotide_Polymorphisms_of_CXCL9_11.3.aspx?casa_token=2kMFCv_y5KcAAAAA:HJXjuc13C4IdQ0jXRz84X8bBYfKwrt3RWyPB1FpyLCOBTq2l4yTRsbYdS8OG9T0O0-hh-nBzVTb-_you33IXjJo 7. Jiménez-Sousa MÁ, Gómez-Moreno AZ, Pineda-Tenor D, Medrano LM, Sánchez-Ruano JJ, Fernández-Rodríguez A, et al. CXCL9-11 polymorphisms are associated with liver fibrosis in patients with chronic hepatitis C: a cross-sectional study. Clin Transl Med [Internet]. 2017 Jul 28;6(1):26. Available from: https://doi.org/10.1186/s40169-017-0156-3 8. Wasmuth HE, Lammert F, Zaldivar MM, Weiskirchen R, Hellerbrand C, Scholten D, et al. Antifibrotic effects of CXCL9 and its receptor CXCR3 in livers of mice and humans. Gastroenterology [Internet]. 2009 Jul;137(1):309–19, 319.e1–3. Available from: http://dx.doi.org/10.1053/j.gastro.2009.03.053 9. Berres M-L, Asmacher S, Lehmann J, Jansen C, Görtzen J, Klein S, et al. CXCL9 is a prognostic marker in patients with liver cirrhosis receiving transjugular intrahepatic portosystemic shunt. J Hepatol [Internet]. 2015 Feb;62(2):332–9. Available from: http://dx.doi.org/10.1016/j.jhep.2014.09.032 10. Shen J, Gao J, Chen C, Lu H, Hu G, Shen J, et al. Antifibrotic role of chemokine CXCL9 in experimental chronic pancreatitis induced by trinitrobenzene sulfonic acid in rats. Cytokine [Internet]. 2013 Oct;64(1):382–94. Available from: http://dx.doi.org/10.1016/j.cyto.2013.05.012 11. O’Brien JC, Rainwater YB, Malviya N, Cyrus N, Auer-Hackenberg L, Hynan LS, et al. Transcriptional and Cytokine Profiles Identify CXCL9 as a Biomarker of Disease Activity in Morphea. J Invest Dermatol [Internet]. 2017 Aug;137(8):1663–70. Available from: http://dx.doi.org/10.1016/j.jid.2017.04.008 12. Mertens JS, de Jong EMGJ, Pandit A, Seyger MMB, Hoppenreijs EPAH, Thurlings RM, et al. Regarding “Transcriptional and Cytokine Profiles Identify CXCL9 as a Biomarker of Disease Activity in Morphea.” J Invest Dermatol [Internet]. ncbi.nlm.nih.gov; 2018 May;138(5):1212–5. Available from: http://dx.doi.org/10.1016/j.jid.2017.11.032 13. Hasegawa M, Fujimoto M, Matsushita T, Hamaguchi Y, Takehara K, Sato S. Serum chemokine and cytokine levels as indicators of disease activity in patients with systemic sclerosis. Clin Rheumatol [Internet]. 2011 Feb;30(2):231–7. Available from: http://dx.doi.org/10.1007/s10067-010-1610-4 14. Rabquer BJ, Tsou P-S, Hou Y, Thirunavukkarasu E, Haines GK 3rd, Impens AJ, et al. Dysregulated expression of MIG/CXCL9, IP-10/CXCL10 and CXCL16 and their receptors in systemic sclerosis. Arthritis Res Ther [Internet]. 2011 Feb 8;13(1):R18. Available from: http://dx.doi.org/10.1186/ar3242 15. Liu X, Mayes MD, Tan FK, Wu M, Reveille JD, Harper BE, et al. Correlation of interferon-inducible chemokine plasma levels with disease severity in systemic sclerosis. Arthritis & Rheumatism [Internet]. 2013;65(1):226–35. Available from: https://onlinelibrary.wiley.com/doi/abs/10.1002/art.37742 16. Hintermann E, Bayer M, Pfeilschifter JM, Luster AD, Christen U. CXCL10 promotes liver fibrosis by prevention of NK cell mediated hepatic stellate cell inactivation. J Autoimmun [Internet]. 2010 Dec;35(4):424–35. Available from: http://dx.doi.org/10.1016/j.jaut.2010.09.003 17. Tager AM, Kradin RL, LaCamera P, Bercury SD, Campanella GSV, Leary CP, et al. Inhibition of pulmonary fibrosis by the chemokine IP-10/CXCL10. Am J Respir Cell Mol Biol [Internet]. 2004 Oct;31(4):395–404. Available from: http://dx.doi.org/10.1165/rcmb.2004-0175OC 18. Jiang D, Liang J, Campanella GS, Guo R, Yu S, Xie T, et al. Inhibition of pulmonary fibrosis in mice by CXCL10 requires glycosaminoglycan binding and syndecan-4. J Clin Invest [Internet]. 2010 Jun;120(6):2049–57. Available from: http://dx.doi.org/10.1172/JCI38644 19. Cardarelli S, Facco M, Fittà C, Del Rosso A. CXCL11 in bronchoalveolar lavage fluid and pulmonary function decline in systemic sclerosis. Clinical and [Internet]. 2012; Available from: https://www.academia.edu/download/45798612/CXCL11_in_bronchoalveolar_lavage_fluid_a20160520-15769-1pwsbty.pdf 20. Burdick MD, Murray LA, Keane MP, Xue YY, Zisman DA, Belperio JA, et al. CXCL11 attenuates bleomycin-induced pulmonary fibrosis via inhibition of vascular remodeling. Am J Respir Crit Care Med [Internet]. 2005 Feb 1;171(3):261–8. Available from: http://dx.doi.org/10.1164/rccm.200409-1164OC 21. Sulpice E, Contreres J-O, Lacour J, Bryckaert M, Tobelem G. Platelet factor 4 disrupts the intracellular signalling cascade induced by vascular endothelial growth factor by both KDR dependent and independent mechanisms. Eur J Biochem [Internet]. 2004 Aug;271(16):3310–8. Available from: http://dx.doi.org/10.1111/j.1432-1033.2004.04263.x

Morphea, or localized scleroderma, is an inflammatory fibrosing disease of the dermis and underlying tissue. Several studies have identified CXCR3 ligands as positively correlating with disease severity and activity in patients ^55– 57^. Systemic sclerosis, or scleroderma, also exhibits upregulation of CXCR3 ligands that correlates with disease severity ^58^. Preliminary studies from our laboratory support a pro-fibrotic role of CXCL9 in the skin: CXCL9-/- mice are protected from bleomycin-induced skin fibrosis, and *in vitro* treatment of mouse and human fibroblasts with CXCL9 induces transcription of collagen 1a1 (col1a1); these data are available on a preprint server and are currently undergoing peer review ^59^.

CXCL10 has pro-fibrotic effects in the liver, where it prevents NK cells from inactivating hepatic stellate cells ^60^. CXCL10-/- mice and WT mice treated with anti-CXCL10 antibody are protected from carbon tetrachloride-induced liver fibrosis. Hepatic stellate cells upregulate CXCR3 in response to carbon tetrachloride, and CXCL10 induces their migration but not proliferation. CXCL10 also mediates T and B cell aggregates in lymphoid tissue, which are essentially absent in CXCL10-/- mice ( Table 1).

## Antifibrotic roles of CXCR3 ligands

While CXCL9 has pro-fibrotic effects in renal and skin tissue, CXCL9 has direct angiostatic and antifibrotic effects in experimental models of pancreas and liver fibrosis. In the trinitrobenzene sulfonic acid (TNBS) induced-pancreatitis rat model, administration of anti-CXCL9 antibody worsened fibrosis, whereas administration of recombinant CXCL9 improved fibrosis, as assessed by trichrome staining and hydroxyproline assay ^61^ ( Table 1). *In vitro* stimulation of pancreatic stellate cells with CXCL9 downregulated TGFβ1 and col1a1 production by confocal microscopy. Of note, antibody and recombinant CXCL9 were administered subcutaneously (s.c.) to rats in this model. We hypothesize that this route of administration may have pulled inflammatory infiltrates away from the gastrointestinal (GI) tract and towards the skin, considering there was 1.5ng/mL CXCL9 in serum and approximately 30µg was administered s.c. daily (assuming average weight of 300g/rat at a dose of 100 μg/kg body weight).

In the carbon tetrachloride-induced liver fibrosis model, CXCR3-/- mice exhibited augmented liver damage at 24h ^62^. Follow-up studies from the same laboratory used mice treated exogenously with CXCL9, which reduced the severity of liver fibrosis as assessed by Sirius red staining, hydroxyproline assay, and α-SMA expression ^63^ ( Table 1). *In vivo* CXCL9 treatment also inhibited angiogenesis as assessed by CD31 staining and ultrasound visualization of liver perfusion. However, CXCL9 treatment did not impact the number of Th1-polarized, IFN-γ-positive cells in the liver amongst treatment groups. Treatment of endothelial cells *in vitro* with CXCL9 was able to inhibit VEGF-mediated proliferation and migration via PLCγ, JNK and ERK. *In vitro* treatment of hepatic stellate cells reduced TGFβ and col1a1 by protein and RNA ^64^.

While CXCL10 has profibrotic effects in the liver, CXCL10 limits lung fibrosis in the murine model of bleomycin-induced pulmonary fibrosis ( Table 1). CXCR3-/- and CXCL10-/- mice display exaggerated pulmonary fibrosis after bleomycin administration, and transgenic mice overexpressing CXCL10 are protected from bleomycin-induced mortality ^65, 66^. Bleomycin did not alter the T cell cytokine milieu in CXCL10-/- mice, weakening the support for the idea that CXCL10 might limit fibrosis by skewing T cell polarization to the Th1 phenotype as demonstrated in hepatitis models. CXCL10 also did not decrease lung tissue-derived angiogenic activity and von Willebrand Factor expression after bleomycin delivery, despite that angiogenesis is considered a rate-limiting step in the development of pulmonary fibrosis. CXCR3 mRNA, but not protein, was detected in lung fibroblasts. Rather, direct interaction of the heparin-binding domain of CXCL10 and syndecan-4 on the lung interstitial compartment inhibits fibroblast recruitment, TGFβ signaling and subsequent fibrosis ^67, 68^. Similar findings were reported in myocardium, which required CXCL10 fibroblast responses through proteoglycans ^69^, and urethral fibrosis, in which CXCL10 signaling interfered with profibrotic TGFβ signaling ^70^.

Similar to CXCL10, CXCL11 attenuates lung fibrosis in the bleomycin mouse model and inhibits angiogenesis in the corneal micropocket assay ^71^ ( Table 1). A double-blind, placebo controlled study of 330 idiopathic pulmonary patients treated with subcutaneous IFN-γ 1b treatment exhibited increased CXCL11 in bronchoalveolar lavage fluid and plasma, with concomitant decreased elastin ^72^. The pro- and anti-fibrotic roles of the CXCR3 ligands in different organs are summarized in Table 2.

**Table 2.** Comparison of CXCR3 ligand actions in organ fibrosis.

	CXCL9	CXCL10	CXCL11
**Profibrotic**	Heart, Kidney, Skin	Heart, Liver	?
**Antifibrotic**	Liver, Pancreas	Lung	Lung

## Potential therapeutic manipulations of CXCR3 for the treatment of fibrosis

To select how to manipulate CXCR3 and/or its ligands for the treatment of fibrosis, it is our opinion that the suspected cell-of-origin in the fibrotic response and the level of angiogenesis during fibrogenesis need to be assessed. Based on the evidence discussed above, we hypothesize that fibrosing disorders primarily mediated by pericyte-type cells that require ERK signaling and exhibit more angiogenesis as a disease feature would be more dependent upon CXCL10, and fibrosing disorders primarily mediated by fibroblast or myofibroblast-type cells that require AKT and JAK signaling and exhibit less vascular involvement would be more dependent upon CXCL9. For example, hepatic fibrosis has prominent vascular changes and is driven by hepatic stellate cells and CXCL10, whereas morphea has a low incidence of vascular changes and is driven by fibroblasts/myofibroblasts and CXCL9. GI organs, in which fibrosis is driven by pericytes, also generally seem to use CXCL9 for protective responses, whereas lung and skin, in which fibrosis is driven by fibroblast subsets, use CXCL10 for protective responses. Nuances in the signaling pathways, the relative chemokine responsiveness, as well as potential coreceptors, will need to be addressed in future studies. Technologies such as single cell RNA sequencing and proteomics may ultimately help resolve the heterogeneity of chemokine receptor and coreceptor expression, as well as preferential signaling pathway usage.

The first potential class of small molecules that could be used to disrupt CXCR3-mediated inflammatory fibrosis are JAK inhibitors. We and others have shown that JAK inhibitors prevent fibrosis in mice ^73– 75^, and demonstrated efficacy in our case studies of human morphea patients who were recalcitrant to standard therapies ^73, 76^. In our study of intradermal bleomycin injection in mice and human morphea tissue, we observed p-STAT1 and p-STAT3 activation in both immune infiltrates and cells with fibroblast morphology ^73^. Notably, STAT1, STAT3 and STAT5 have predicted binding sites in the collagen 1a1 (col1a1) promoter and enhancer regions (GeneCards), which may account for our observation that JAK inhibitors were able to suppress col1a1 transcription by human and mouse fibroblasts *in vitro* ^73^. We also noted that the JAK 1/2 inhibitor ruxolitinib yielded a slightly better p value than the JAK 3>>1>2 inhibitor tofacitinib for inhibition of dermal thickening in the intradermal bleomycin mouse model. These data are in agreement with previously published studies examining JAK2 as a driver of fibrosis in scleroderma fibroblasts and a bleomycin mouse model ^74^. Zhang *et al* demonstrated that following long-term selective inhibition of JAK2, JAK2 may be transphosphorylated by JAK1 to mediate fibrosis ^77^, supporting the use of a combination JAK1/2 inhibitor for treatment of fibrosis. It is interesting to note that ruxolitinib was originally FDA approved for myelofibrosis ^78^, and patients receiving ruxolitinib therapy often resolve fibrosis ^79^. While this is encouraging for potential repurposing of ruxolitinib for other fibrosing diseases, we would caution that careful tapering and monitoring is needed to prevent potential rebound effects ^80, 81^. Cessation of ruxolitinib can cause hyperphosphorylation of JAK2, increasing inflammation and subsequent fibrosis ^82^. Selecting a JAK1/2 inhibitor with a longer half-life, such as baricitinib ^83^, might be a safer option for patients who are tapering.

The second potential class of therapeutics would be agonist peptides to mimic the antifibrotic role of CXCL10 for lung fibrosis. As suggested by Tager and Jiang *et al*, maintaining heparin binding but excluding CXCR3 binding would mitigate potential toxicities related to T cell recruitment ^65, 67^. CXCL10-based therapeutics might also prove useful for improving lung fibrosis and function in patients recovering from infectious lung disease, particularly Sars-CoV2 infection/COVID-19 disease ^84^. Smith *et al* recently reported biased agonists of CXCR3 that can differentially mediate inflammation and migration of immune cells which they examined in the context of contact hypersensitivity in skin ^14^, providing a basis for the feasibility of this approach.

The third potential class of therapeutics would be agents that inhibit the pro-fibrotic signaling events mediated by CXCR3 ligands, such as CXCL9 in Th1/IFNγ-driven kidney disease or morphea. These approaches could include anti-CXCL9 blocking/neutralizing antibodies, CXCL9 siRNA, or antagonistic peptide ligands. Of note, antibody neutralization of CXCL10 for treatment of hepatic fibrosis may be challenging, as CXCL10 antibodies neutralize the free form and not endothelial-bound chemokine ^85^. Similar challenges may arise when attempting to neutralize CXCL9 with antibody, as would anti-drug antibody responses.

A fourth class of therapeutics could leverage the cell- or organ-specific context of chemokine expression. For example, stimulating γδ T-cells to produce CXCL10 in the lung could have therapeutic benefits in pulmonary fibrotic disease. Inhibiting macrophage production of CXCL9 in the skin could prevent collagen deposition in morphea. Drawing immune infiltrates away from the pancreas and towards the skin could reset the fibrotic process, as in the TNBS-induced rat model. Agents are in development to target specific cell types, such as antibody-drug conjugates ^86^ some with cleavable linkers ^87^, bispecific antibodies ^88, 89^ and nanoparticles ^90^, which can be preferentially phagocytosed by antigen presenting cells of the immune system. These could be leveraged to achieve the aforementioned goals of stimulating CXCL10 or inhibiting CXCL9 production by key cell types. Different drug delivery routes and systems may also help accomplish the goal of drawing immune cells away from the pancreas or other organs, with cutaneous administration via creams, injections or microneedle patches ^91^ helping to achieve a high local concentration.

A fifth class of therapeutics could leverage existing enzymatic cleavage of CXCR3 ligands. Recombinant peptides lacking amino-terminal amino acids can exert angiostatic effects, while inhibiting CXCR3-mediated migration. Administration of bioactive CD26 and/or CD13, or inhibitors of these enzymes, may modulate fibrotic processes in specific organs or diseases. CD26 inhibitors have been reported to reduce or prevent fibrosis in models of myocardial fibrosis, lung fibrosis and kidney fibrosis ^92– 94^, and a CD13 inhibitor improved fibrosis in a mouse model of silica-induced lung fibrosis ^95^.

Last, combination therapies targeting both prevention of inflammation and fibrosis in addition to promoting tissue remodeling will likely provide the best therapeutic outcome for fibrosis patients ^96^. Tissue remodeling will ultimately allow for breakdown of fibrotic plaques and better disease outcomes, which could be achieved through agonists or inducers of matrix metalloproteinases (MMPs) or antagonists of tissue inhibitors of MMPs (TIMPs).

## Conclusion

The differential impact of CXCR3 and its ligands on tissues depends on disparate signaling pathways involving multiple cell types and potential coreceptors; the nuances of which should be addressed in future research involving single cell RNA sequencing and proteomics. As we have examined the known fibrotic and antifibrotic roles of CXCR3 and its ligands, we suggest that future therapeutic options should be centered around the suspected cell-of-origin in the fibrotic response, tissue-specific signaling factors and the degree of angiogenesis is during fibrosis. Likely, a combination of these therapies will have the best potential to ameliorate symptoms of patients with fibrosing diseases.

## Data availability

No data are associated with this article.
