# Supplementary material for: Potential therapeutic manipulations of the CXCR3 chemokine axis for the treatment of inflammatory fibrosing diseases
Source: F1000Res. 2020 Oct 5;9:1197. [Version 1] doi: 10.12688/f1000research.26728.1 (PMC7590900; doi:10.12688/f1000research.26728.1)
Supplement: Peer review history

# Peer review: Reviewer response for version 1

**DOI:** 10.5256/f1000research.29512.r72472

**Published:** 2020-10-22

**Authors:** Patricia A. Pioli

**Affiliations:** 1 Department of Microbiology and Immunology, Geisel School of Medicine, Lebanon, NH, USA

This report provides an excellent summary of the role of CXCR3 and associated ligands (CXCL9, 10 and 11) in fibrosis. Significantly, these mediators have been reported to have both pro- and anti-fibrotic effects, which potentially confounds their importance in the regulation of fibrotic activation. One of the strengths of this report is that it provides an explanation for these ostensibly opposing effects by emphasizing the importance of cell and tissue context and the role of angiogenesis. Indeed, rather than serving redundant functions, it is likely that the relative contribution of these chemokines is dictated by the extent of vascularization and the cell type responsible for mediating tissue damage. As suggested by the authors, the use of combination therapies is likely to generate the most therapeutic benefit to patients, and the last section of this review does an excellent job of summarizing treatment targets. Although beyond the scope of this current article, future reviews may investigate the utility of specifically targeting immune subpopulations as sources of these chemokines.

Is the topic of the opinion article discussed accurately in the context of the current literature?

Yes

Are arguments sufficiently supported by evidence from the published literature?

Yes

Are all factual statements correct and adequately supported by citations?

Yes

Are the conclusions drawn balanced and justified on the basis of the presented arguments?

Yes

Reviewer Expertise:

macrophage activation  in fibrotic disease, including systemic sclerosis

I confirm that I have read this submission and believe that I have an appropriate level of expertise to confirm that it is of an acceptable scientific standard.

# Peer review: Reviewer response for version 1

**DOI:** 10.5256/f1000research.29512.r72473

**Published:** 2020-10-16

**Authors:** Christian D. Sadik

**Affiliations:** 1 Department of Dermatology, Allergy, and Venereology, University of Lübeck, Lübeck, Germany; 2 Center for Research on Inflammation of the Skin (CRIS), Lübeck, Germany

The review focuses on the role of CXCR3 and its ligands in fibrotic processes. It starts with an excellent overview on the complex receptor pharmacology of CXCR3 and continues to exhaustively summarize and discuss the role of CXCR3 in the fibrotic processes in different organs.

 The review is very illustrative including excellent figures. The current state of knowledge on CXCR3 and its role in fibrotic processes is comprehensively summarized and discussed in a balanced manner. All in the article are supported by the literature, which is adequately cited.

Is the topic of the opinion article discussed accurately in the context of the current literature?

Yes

Are arguments sufficiently supported by evidence from the published literature?

Yes

Are all factual statements correct and adequately supported by citations?

Yes

Are the conclusions drawn balanced and justified on the basis of the presented arguments?

Yes

Reviewer Expertise:

My areas of research are innate immunity, immomediators, immunopharmacology, and autoimmune and autoinflammatory diseases

I confirm that I have read this submission and believe that I have an appropriate level of expertise to confirm that it is of an acceptable scientific standard.

# Response

**Published:** 2020-10-19

**Authors:** Jillian Richmond

**Affiliations:** UMass Medical School, USA

We thank Dr. Sadik for taking the time to review our opinion article.
